# Genome-wide association study of drought tolerance and biomass allocation in wheat

**DOI:** 10.1371/journal.pone.0225383

**Published:** 2019-12-04

**Authors:** Isack Mathew, Hussein Shimelis, Admire Isaac Tichafa Shayanowako, Mark Laing, Vincent Chaplot

**Affiliations:** 1 African Centre for Crop Improvement, University of KwaZulu-Natal, School of Agricultural, Earth and Environmental Sciences, Pietermaritzburg, South Africa; 2 University of KwaZulu-Natal, School of Agricultural, Earth and Environmental Sciences, Pietermaritzburg, South Africa; 3 Sorbonne Universities, UPMC, IRD, CNRS, MNHN, Laboratoire d’Océanographie et du Climat: Expérimentations et approches numériques (LOCEAN), IPSL, Paris, France; Mahatma Phule Krishi Vidyapeeth College of Agriculture, INDIA

## Abstract

Genome wide association studies (GWAS) are important in discerning the genetic architecture of complex traits such as biomass allocation for improving drought tolerance and carbon sequestration potential of wheat. The objectives of this study were to deduce the population structure and marker-trait association for biomass traits in wheat under drought-stressed and non-stressed conditions. A 100-wheat (*Triticum aestivum* L.) genotype panel was phenotyped for days to heading (DTH), days to maturity (DTM), shoot biomass (SB), root biomass (RB), root to shoot ratio (RS) and grain yield (GY). The panel was sequenced using 15,600 single nucleotide polymorphism (SNPs) markers and subjected to genetic analysis using the compressed mixed linear model (CMLM) at false discovery rate (FDR < 0.05). Population structure analysis revealed six sub-clusters with high membership ancestry coefficient of ≤0.65 to their assigned sub-clusters. A total of 75 significant marker-trait associations (MTAs) were identified with a linkage disequilibrium threshold of 0.38 at 5cM. Thirty-seven of the MTAs were detected under drought-stressed condition and 48% were on the B genome, where most quantitative trait loci (QTLs) for RB, SB and GY were previously identified. There were seven pleiotropic markers for RB and SB that may facilitate simultaneous selection. Thirty-seven putative candidate genes were mined by gene annotation on the IWGSC RefSeq 1.1. The significant MTAs observed in this study will be useful in devising strategies for marker-assisted breeding for simultaneous improvement of drought tolerance and to enhance C sequestration capacity of wheat.

## Introduction

Bread wheat (*Triticum aestivum* L., 2n = 6x = 42, AABBDD) is a commodity crop with a global harvested area of over 210 million hectares [1]. It is a source of food for over 2.5 billion people worldwide [2]. Wheat production and productivity is challenged by numerous biotic and abiotic stresses. Among the major abiotic constraints is recurrent drought driven by climate change. In addition, the inherently low-fertile soils, notably in sub-Sahara Africa, exacerbate the impact of drought stress resulting in higher wheat yield losses compared to other regions [3].

One mechanism by which plants respond to environmental stresses is to adjust their biomass allocation [4]. Therefore, exploiting the phenotypic plasticity present in biomass allocation encompassing the root system of modern wheat cultivars has been proposed as a method to improve drought resilience and yield potential [5]. Enhancing biomass allocation to roots will improve drought tolerance by increasing the moisture extraction capability, while promoting soil C input via root exudation and turnover [6]. However, simultaneous improvement for drought tolerance and C sequestration has not been pursued in crop breeding programs [7], particularly in cereals such as wheat where breeding for high grain yield is the primary objective. In addition, there has been few studies on genetic analysis of root traits because they are difficult to phenotype and require routine destructive sampling [8]. Most importantly, progress in breeding for drought tolerance is slow because of its low heritability and polygenic nature. Identifying the underlying genetic loci for root, shoot and grain biomass under contrasting environments will enable marker-assisted selection to improve selection efficiency [9] and to accelerate development of cultivars with optimal biomass allocation for drought tolerance and C sequestration.

The advent of next generation sequencing (NGS) and genotyping by sequencing (GBS) technologies has provided a means for examining genetic diversity and discovering novel markers [10]. Marker systems such as simple sequence repeats (SSR) and SNPs have been used successfully to elucidate the genetic attributes of complex traits in wheat [11; 12; 13]. Micro-array-based diversity array technology sequencing (DArTseq)-derived single nucleotide polymorphisms (SNPs) have become increasingly important in genome-wide association studies (GWAS) [14]. The DArTseq-derived SNPs have been used extensively on genetic studies of wheat [15; 16; 17]. These markers are reproducible and provide a powerful means to identify genetic variation and genetic makeup at large number of analogous genomic loci such as present in hexaploid wheat. This enables breeders to deduce genetic diversity and genomic loci controlling economic traits through association mapping [18] and to identify QTLs responsible for traits such as drought tolerance and high C sequestration.

It is important to deduce associations between markers and traits to improve efficiency of conventional breeding methods. Genetic markers for several agronomic traits have been identified [17] but genetic control of biomass allocation to yield, shoot and root components remains less investigated despite its important implications for drought tolerance. The genomic loci associated with improved drought tolerance due to high rooting capacity have been elucidated in other crop species such as soya [19], rice [20] and chickpea [21]. The identification of genomic loci for root traits in wheat is limited by the difficulties associated with root phenotyping and the relatively large size and complexity of the wheat genome [22; 23]. However, the scarcity of genetic markers and limited studies on marker-trait associations for biomass allocation and related traits impede the use of marker-assisted selection (MAS) in developing breeding populations for drought tolerance and C sequestration in wheat. Hence, studies on biomass allocation to roots, shoots and grains are required to identify reliable and stable markers. Therefore, the objective of this study was to deduce marker-trait associations for biomass allocation and yield-related traits in a diverse population of wheat genotypes for future marker-assisted breeding to improve drought tolerance and enhanced C sequestration capacity of wheat.

## Material and methods

### The germplasm

A panel of 100 wheat genotypes were evaluated. The bread wheat genotypes included 95 drought and heat tolerant genotypes initially acquired from the International Maize and Wheat Improvement Center (CIMMYT). These genotypes were purposefully selected for their genetic divergence and breeding history for drought tolerance. The remainder of the bread wheat genotypes were two local checks and two commercial cultivars adapted to temperate climates. The temperate commercial cultivars were included in the study to widen the genetic diversity for rooting ability. The commercial varieties adapted to temperate climates have twice the rooting capacity of wheat grown in warmer winters [24]. The details of the germplasm are presented in S1 Table.

### Phenotyping

Test materials were phenotyped involving two experiments under drought-stressed and non-stressed conditions. The two experiments corresponded to four test environments that included two experiments each under drought-stressed and non-stressed conditions in the greenhouse and field. The greenhouse experiments were conducted at controlled environment facility of the University of KwaZulu-Natal (UKZN), while the field trial was conducted at Ukulinga Farm of the UKZN (LAT: 29.667 LON: 30.406 and ALT: 811m) between 2016 and 2018. In the greenhouse experiment, 100 genotypes were sown in October in 2016, while the second experiment was established in May 2017. Both trials were conducted using a 10×10 alpha lattice design with 2 replications. The greenhouse provided shelter against rainfall and irrigation was provided via an automated drip irrigation system inserted directly into individual pots. Fertilizer was also applied through automated drip irrigation at a rate of 300 kg N ha^-1^ and 200 kg P_2_O_5_ ha^-1^. The different water regimes were initiated 6 weeks after planting to ensure good establishment but also to ensure early exposure of all growth stages to drought. In the non-stress condition, the plants were watered to field capacity (FC) whenever average soil water content fell to 80% of FC, while in the drought stress conditions volumetric soil water content was allowed to drop to 30% of FC before watering to FC. The soil water content was monitored by a soil moisture probe and random weighing of the pots. The two watering treatments were maintained until maturity (~120 days).

The field experiments was established in May 2017 following a similar design. The soil surface was covered by a custom made plastic, which acted as a mulch to prevent rain water from entering into the soil. Basal fertilizer composed of nitrogen (N), phosphorous (P) and potassium (K) was applied at a rate of 120:30:30 kg ha^-1^ (N:P:K). Other agronomic practices were as per normal wheat production practice in South Africa [25]. Irrigation was applied through a drip irrigation system with the aim to maintain soil water content at FC in the well-watered regime. Under the drought stress treatment, irrigation was withheld 5 weeks after crop emergence until just before signs of permanent wilting were observed upon which irrigation was reinstated. This differs from the 80 and 30% FC soil water regimes maintained in the greenhouse because it is more difficult to determine field capacity and regulate soil water content appropriately under field conditions compared to a controlled greenhouse environment. During the field experiment, irrigation was withheld before anthesis to induce drought stress in a way that simulated *in situ* field wheat production. Amount of water applied and prevailing temperatures were recorded for the period to determine the extent of drought stress [26]. The following phenotypic traits were assessed: the number of days to 50% heading (DTH) and number of days to 50% maturity (DTM) were counted from date of planting, plant height (PH) expressed in centimeter and spike length (SL, cm) were measured with a calibrated ruler, shoot biomass (SB, grams per m^2^), root biomass (RB, grams per m^2^), thousand kernel weight (TKW, g 1000^−1^ seeds) and grain yield (GY grams per m^2^) were weighed on a laboratory precision digital scale at maturity while root to shoot ratio (RS) were derived from RB and SB accordingly and the number of kernels per spike (KPS) were counted after shelling.

Phenotypic data were subjected to the Shapiro-Wilk test for normality before analysis of variance in Genstat 18^th^ edition [27]. Variance components were calculated in Genstat 18th edition using the general linear model [27] where the environments and water regimes were considered to have fixed effects while genotype effects were treated as random following [28]. General statistics including means, standard error, and coefficient of variation for the phenotypic data were computed in Genstat 18^th^ [27]. The associations among the phenotypic traits were tested using Pearson correlations. Broad sense heritability (H^2^) estimates were calculated from phenotypic variance (σ^2^_*p*_) and the genotypic variance (σ^2^_*g*_) according to [29] as follows:

$$H=\frac{\delta^{2}g}{\delta^{2}p}$$

Where δ^2^*p* = δ^2^g+ δ^2^ge/e + δ^2^e/re

Where δ^2^p = phenotypic variance, δ^2^g = genotypic variance, δ^2^ge = genotype × environment interaction variance, δ^2^e = residual variance while r = number of replications and e = number of environments. The inclusion of three environments allows for an effective evaluation of quantitative traits and ensures precision in estimating heritability values [30; 31].

### Genotyping

The 100 genotypes were planted in the greenhouse in seedling trays. Genomic DNA was extracted from leaves of 3-week old seedlings. The DNA was extracted using CTAB method [32]. After extraction, the nucleic acid concentration and purity of the DNA was checked using a NanoDrop 2000 spectrophotometer (ND- 2000 V3.5, NanoDrop Technologies, Inc.) before being shipped to Diversity Arrays Technology (DArT) Pty Ltd, Australia for whole genome sequencing on the DArTseq platform. Whole-genome genotyping for the 100 wheat genotypes was carried out on the platform developed by [33] using 28,356 DArT markers. The markers were integrated into a linkage map by inferring marker order and position from the consensus DArT map. The mean polymorphic information of the markers was 0.16 and ranged between 0.0 and 0.50 with a reproducibility index of 0.93.

#### DArTseq SNP filtering

All the individuals were genotyped using 28,356 silico DArT markers assigned to 21 chromosomes. A total of 15,600 informative DArTseq-derived SNP markers and 99 genotypes were used after data imputation where SNP loci and individuals with >20% missing data and rare SNPs with <5% minor allele frequencies (MAF) were pruned from the data before analysis as previously described by [16].

#### Population structure

The population structure of the 99 genotypes was assessed using the Bayesian clustering method in STRUCTURE version 2.3.4 [34]. A 10,000 burn-in period and 10,000 Markov Chain Monte Carlo (MCMC) iterations were used to derive the population structure based on 15,600 DArTseq-derived SNP markers distributed across the wheat genome. The K-value was set between 1 and 10 to generate the number of subpopulations in the accessions. The best K-value for estimating a suitable population size for the dataset was determined by the K-value with the highest likelihood to reduce the risk of false positive associations [35]. The optimal number of clusters and sub-clusters in the population were determined by the Evanno method based on ΔK and the highest median values of Ln(Pr Data) in CLUMPAK [36].

#### Association mapping

A total of 15,600 DArTseq derived SNP markers and best linear unbiased predictors (BLUPs) for the phenotypic traits measured under different environments were used to determine marker-trait associations among the 99 accessions in the population. This panel formed a core set of new wheat introductions for drought tolerance breeding in South Africa. Usually large number of accessions are used but population sizes between 60 and 150 have been used successfully in previous studies [37; 38; 39; 40]. The information obtained from this GWAS would provide useful baseline information since there are very few studies elucidating genetic control of biomass allocation and its impact on drought tolerance. Prior to conducting GWAS analysis, the phenotypic data collected from each of the experiments were analyzed using the nlme package in R to generate best linear unbiased predictors (BLUPs). Genotype was fitted as a fixed effect and environment were fitted as random effect. The BLUPs for each genotype were used as input in GWAS analysis to handle the variations among the environments. The BLUPs would allow to make unbiased adjustment for fixed effects thus eliminating the need to consider marker trait associations (MTAs) for individual environments. The association mapping was conducted on biomass allocation traits (RB, SB, GY and RS) using a compressed mixed linear model (CMLM) method that factors in both population structure and kinship using the Q + K model where Q = population structure determined by principal component analysis and K = kinship matrix generated in TASSEL 5. The marker-trait association analysis was conducted in the GAPIT program of the R software [41]. The population structure matrix (Q) was fitted as a fixed factor while the kinship matrix (K) was treated as a random factor. The markers were considered as significant for each trait individually at a critical p-value of 1% and false discovery rate of 5%, which was deemed to be highly stringent to reduce the risk of false marker-trait associations (MTAs) [35; 42].

#### Determination of Linkage disequilibrium

The GAPIT program in R software was used to conduct linkage disequilibrium analysis following [41]. Linkage disequilibrium (LD) was based on trait specific genome-wide markers whose positions were specific out of the 15,600 polymorphic markers. The squared allele frequency correlations (R^2^) at p-values <0.001 for each pair of loci were considered to estimate significant linkage disequilibrium. The LD was presented graphically as a heat map constructed using the LDHeatmap package [43] in R [44] based on pairwise R^2^ of SNPs that were significantly associated with each of the traits by plotting the R^2^ values against the genetic distance, in centiMorgans (cM).

#### Putative candidate gene analysis and expression data

Candidate genes overlapping the significant markers were blasted on the IWGSC RefSeq v1.1 using BLASTn function [45]. Genes adjacent to the significant MTAs were identified by the RefSeq v1.0.Gene Ontology (GO) annotation of the potential candidate genes was carried out using Blast2GO pro tool v.3.1.3 [46] and the physical map was visualized on KnetMiner [47; 48]. Subsequently, their molecular function and associated traits were mined from Ensembl plant for *T*. *aestivum*.

## Results

### Phenotyping variation across genotypes and water regimes

The 3-way interaction involving the levels of the following three factors: genotypes, water regime and test environment was significant (p<0.05) for NPT, PH, DTM and RS as revealed by the analysis of variance (ANOVA) (Table 1). The number of days to maturity (DTM) was significantly (p<0.05) affected by the interaction between genotype and water regime. The genotype × test environment interaction effects were significant (p<0.05) on all traits except GY. Individually, the genotype, water regime and test environment effects significantly (p<0.05) affected all traits except TKW. Only six traits, DTH, DTM, RB, SB, RS and GY were considered for further analysis in accordance with the objective of elucidating genetic control of biomass allocation. The DTH and DTM were considered as they affect the phenological development of biomass accumulation and partitioning between vegetative and reproductive organs.

**Table 1:** Mean squares after combined analysis of variance for phenotypic traits of 99 wheat genotypes and a triticale accession evaluated across three test environments under drought-stressed and non-stressed conditions.

SOV	d.f.	DTH	DTM	NPT	PH	RB	RS	SB	TKW	GY
Rep(Env)	2	41.79*	3.32	27.79*	206.82**	12648	0.05**	134719	98.6	148963
Block(Rep)	18	49.48**	43.95**	6.5	142**	38785**	0.01*	908450**	60.7**	332888**
Env	1	65929**	121331**	133**	112698**	2143211**	68.3**	724457871**	9.14	216675286**
Entry	98	295.7**	122.2**	22.3**	515.8**	58678***	0.03**	392742**	117***	268738**
Trt	1	21.72	19983**	4709**	32981**	3590802**	0.0003	80699501**	3115***	42498831**
Env×Entry	96	97.3**	83.7**	10.008*	92.3**	23672*	0.02**	383728**	38.8*	180678
Entry×Trt	97	16.28*	38.06**	8.59	41.79*	21067	0.01*	243278	28.36	122044
Env×Trt	1	4168.9**	302.9**	463**	22816**	53842	0.013	39804625**	1501***	6417019**
Env×Entry×Trt	96	14.51	39.72**	10.592*	44.9**	19388	0.01*	236424	29.79	142093
Residual	368	11.9	15.61	7.669	31.5	16678	0.008	247618	28.6	163088
Total	778	150	226	17.1	316	31316	0.098	1355900	24.8	517915
LSD		4.83	5.4	2.8	5.52	127	0.09	390	5.27	297
CV (%)		4.8	3.29	22.17	7.1	26.19	22.34	32.42	12.1	24.97
se		3.45	3.95	2.76	5.61	79.1	0.09	97.6	2.35	103

SOV = source of variation, DF = degrees of freedom, Rep = replication, Env = Environment, Trt = water regime treatment, DTH = days to heading, DTM = days to maturity, NPT = number of productive tillers, PH = plant height, RB = root biomass weight, SB = shoot biomass weight, RS = root to shoot ratio, TKW = 1000-kernel weight, GY = grain yield, LSD = least significant different at 0.05, CV = coefficient of variation, se = standard error

*, ** and *** = significance level at <0.001, <0.01 and <0.05, respectively.

Days to heading ranged between 40 and 138 with and a mean of 40 (Table 2). Under drought stress, the genotypes matured earlier by an average of 10 days compared to non-stress conditions. Root biomass ranged between 131.3 and 1622.3 g m^-2^ under non-stressed, while it ranged from 64.6 to 735.8 g m^-2^ under drought-stressed conditions. A 32% reduction in mean RB due to drought stress was observed. The lowest shoot biomass was 109 g m^-2^ obtained under stressed condition, while the highest was 1,244 g m^-2^ with improved water availability. The root to shoot ratios varied from a minimum of 0.14 in wheat genotype LM39 and a maximum of 1.45 recorded in triticale. On average, grain yield declined by 48% under drought-stressed condition.

**Table 2:** Summary statistics of biomass and agronomic traits measured in 100 genotypes evaluated in three environments under drought-stressed and non-stressed conditions.

	Non-stressed									Drought-stressed									
	DTH	DTM	NPT	PH	RB	RS	SB	TKW	GY	DTH	DTM	GY	NPT	PH	RB	RS	SB	TKW	GY
Mean	72	125	15	85.5	423.7	0.42	1849.3	45	1088.5	72	115	628.68	10	72.7	289.6	0.42	1218.6	43.9	628.68
Median	75	126	15	82	385.8	0.39	1591.8	45.1	724	72	113	330	10	75	279	0.41	916.5	43.8	330
Minimum	40	94	6	35	131.3	0.04	235.9	26.9	75.1	43	80	67.82	1	23	64.6	0.03	264	23.7	67.82
Maximum	138	148	31	121	1622.3	1.96	8658.3	56.9	4696	132	144	4487.5	19	101.7	735.8	1.09	3775	61.7	4487.5
Quartile 1	61	114	12	69	304.2	0.12	633	41.9	461	66	104	175.36	9	67	206.3	0.11	471.2	41.1	175.36
Quartile 3	84	137	17	104.7	483.9	0.67	2800.3	48.3	1605.5	78	128	1010.79	11	80.7	352.3	0.71	1830.2	47.2	1010.79
St. Dev	14	13	4	19.8	199.5	0.32	1367.1	5	787.5	10	15	556.33	3	12.6	117.4	0.31	801.3	4.9	556.33
SEM	0.7	0.67	0.2	0.99	9.96	0.02	68.36	0.36	39.93	0.53	0.77	28.35	0.13	0.63	5.89	0.02	40.22	0.36	28.35
Skewness	0.16	-0.28	0.52	-0.15	2.14	0.74	0.98	-0.4	1.2	-0.12	-0.18	1.63	0.54	-1.31	0.84	0.11	0.53	-0.14	1.63
Kurtosis	0.64	-1.16	0.85	-1.19	7.02	0.64	1.25	0.71	1.69	2.78	-0.77	5.75	1.01	2.77	1.08	-1.75	-0.82	1.46	5.75

DTH = days to heading, DTM = days to maturity, PH = plant height, RB = root biomass dry weight per m^-2^; SB = shoot biomass dry weight per m^-2^; RS = root to shoot ratio; TKW = 1000-kernel weight, GY = grain weight gm^-2^; SEM = standard error of mean, Std. Dev. = standard deviation

### Population structure analysis

**Fig 1:**
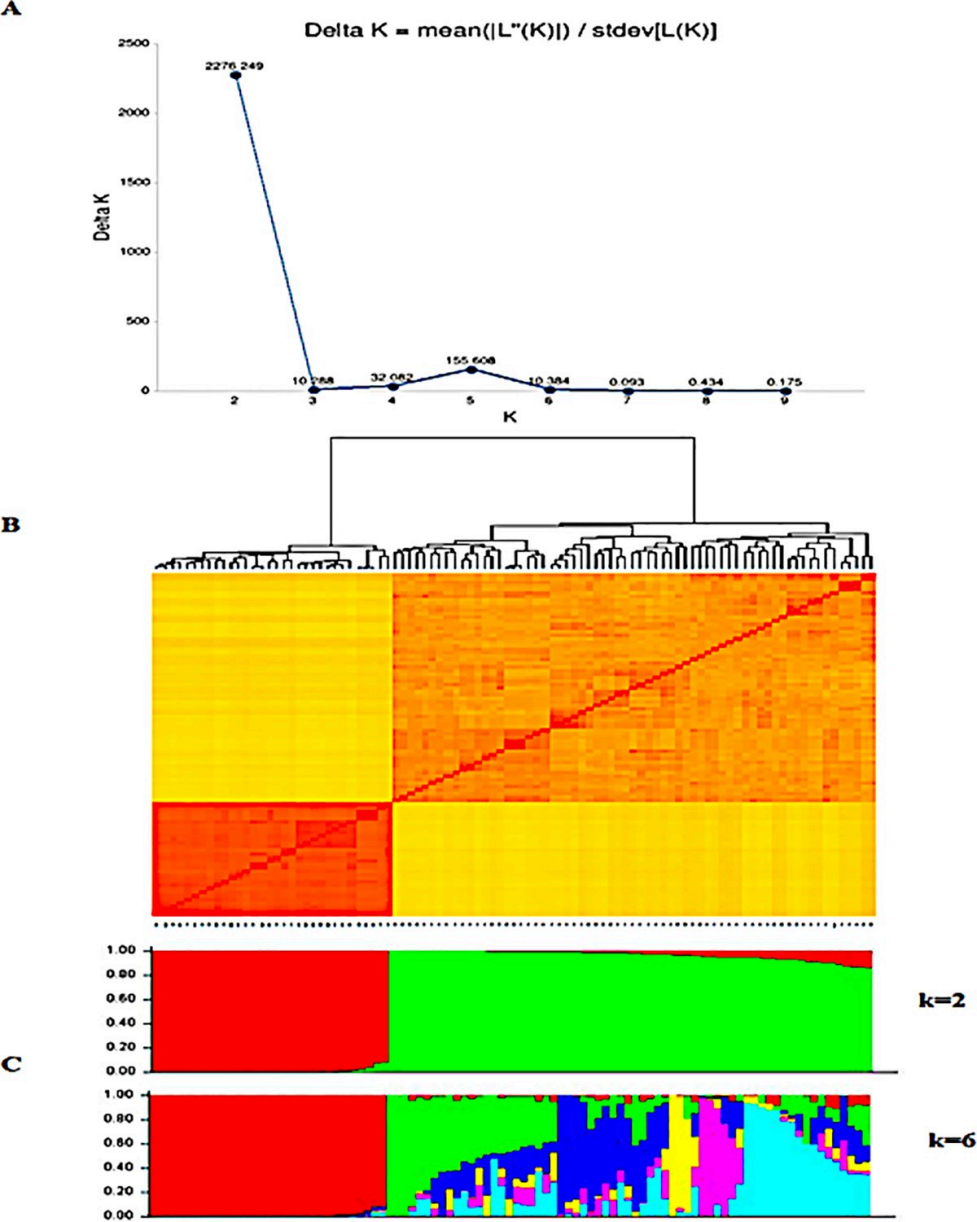
Population structure of 97 wheat genotypes based on 15,600 SNP markers. A. The ΔK determined by the Evanno method showing the stratification of the population into two main clusters. B. The kinship matrix shows the relationship among genotypes. **Fig 2**. Principal component analysis of 97 wheat genotypes based on 15,600 high quality SNPs with MAF > 0.05 using the first three principal components. **A**. The first three principal components accounted for about 47% of variation as indicated on the scree plot. **B**. The genotypes were stratified into two distinct clusters. The six sub-clusters as determined by the highest median values of Ln(Pr Data) based on STRUCTURE. The different colored segment estimate proportion of membership of each genotype to the respective clusters.

**Fig 2:**
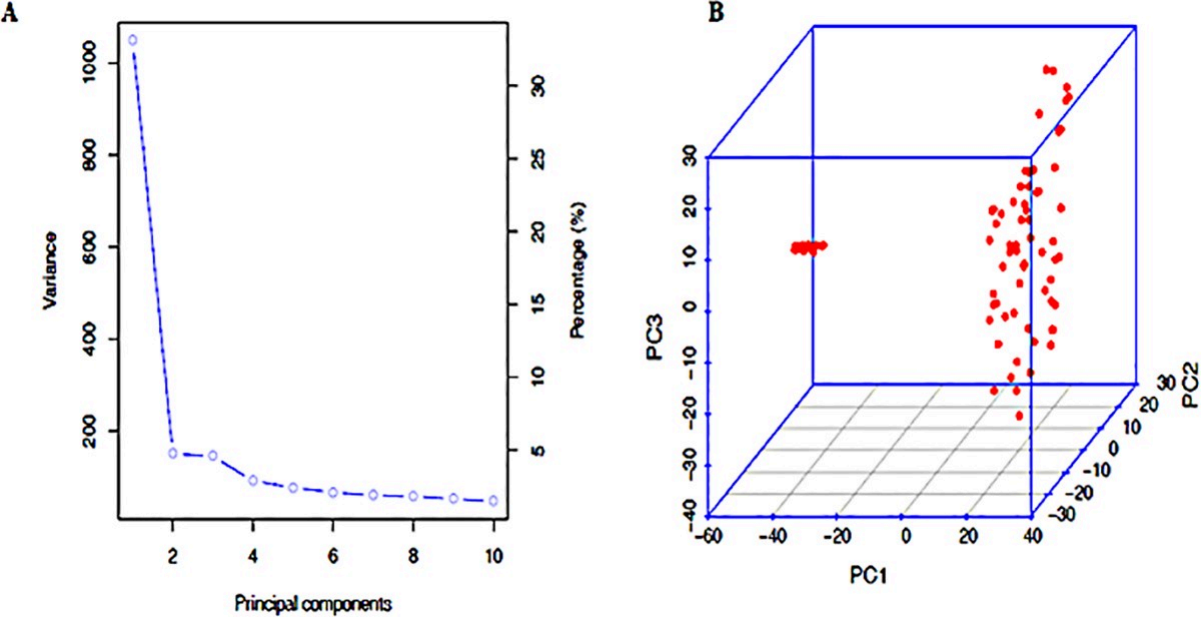
Principal component analysis of 97 wheat genotypes based on 15,600 high quality SNPs with MAF > 0.05 using the first three principal components. A. The first three principal components accounted for about 47% of variation as indicated on the scree plot. B. The genotypes were stratified into two distinct clusters.

The results of STRUCTURE based on markers with MAF>0.05 for the wheat genotypes showed that ΔK was highest at K = 2, showing the presence of two main clusters in the population while the highest median values of Ln (Pr Data) occurred at K = 6, showing that the main clustered could further be divided into six sub-clusters (Fig 1A). The kinship matrix shows a clear stratification of the genotypes into two main clusters and different sub-clusters (Fig 1B). The highest median values of Ln(Pr Data) determined that the population could further be sub-divided into six minor clusters shown by k = 6 (Fig 1C). The principal component analysis (PCA) based on the first three principal components accounted for 47% of the total variation (Fig 2A) and revealed two distinct clusters in the population (Fig 2B). The six minor clusters deduced by the highest median values of Ln(Pr Data) were of different sizes and composition. Membership of all genotypes to a particular sub-cluster was based on at least 65% ancestry. Cluster 1 had the largest membership with 34% of the population, while the smallest was Cluster 4 with only 6% (Table 3). Cluster 1 was comprised entirely of the genotypes from the heat tolerant nursery with code BW, except 2 genotypes LM23 and LM47. Clusters 3 and 6 exhibited the highest level of heterozygosity with an average of 0.23, while the rest of the clusters averaged below 0.15. The mean fixation index (F_st_) ranged between 0.45 and 0.85 among the clusters. The genetic distance among the different populations showed that clusters 1 and 5 were the most divergent with a genetic distance of 0.40 while the shortest distance was between clusters 3 and 6 (S2 Table).

**Table 3:** Genetic clusters and their member genotypes, proportion of membership, expected heterozygosity and the mean values of Fst observed from structure analysis of 97 wheat genotypes and a triticale accession.

Sub-cluster	*Genotypes	% Membership	Expected Heterozygosity	Mean Fixation Index
1	BW120, BW124, BW127, BW140, BW141, BW147, BW148, BW149, BW150, BW151, BW152, BW157, BW159, BW162, BW48, BW71, BW80, LM48, BW142, LM47, BW28, BW58, BW129, BW145, BW103, BW100, BW128, LM23, BW49, BW116, BW63, BW111	34.1	0.14	0.72
2	LM77, LM79, LM90, LM81, LM24, LM98, LM59, LM30, LM14, LM22, LM97, LM27, LM43, LM76, LM16, LM40, LM01, LM44	18.6	0.12	0.73
3	LM33, LM36, LM37, LM32, LM38, LM28, LM31, LM49, LM85, LM39, LM41, LM75, LM26, LM58, LM01, LM42	16.7	0.23	0.49
4	LM51, LM50, LM52, LM86, LM91, LM42	6.0	0.11	0.79
5	LM56, LM57, LM54, LM55, LM20, LM82, LM83, LM42	7.9	0.09	0.85
6	LM96, LM84, LM21, LM18, LM19, LM25, LM29, LM15, LM100, LM80, LM17, LM12, LM16, LM70, LM58, LM83	16.7	0.24	0.45

*the description of genotypes is provided in supplementary table

### Marker-trait associations under different water regimes

**Fig 3:**
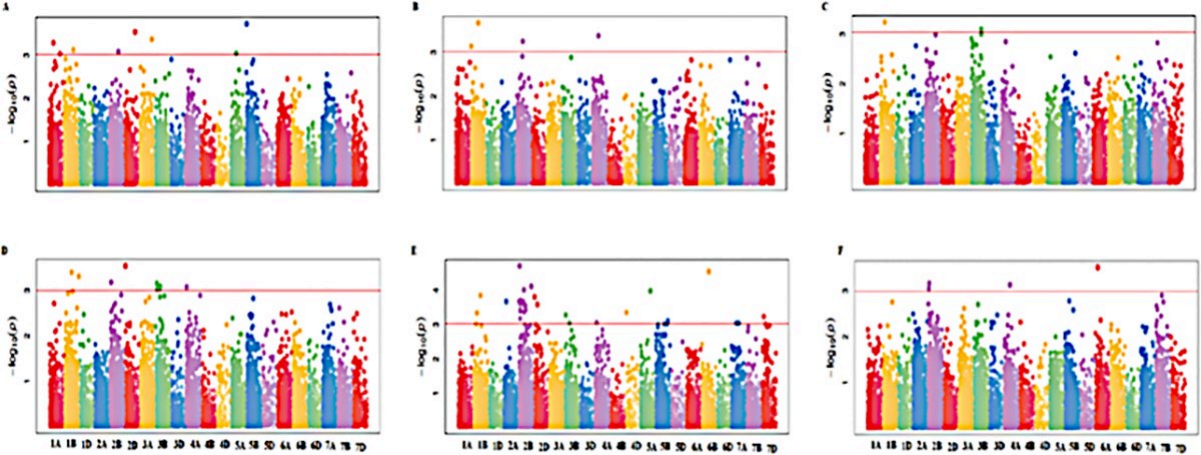
Manhattan plots showing SNP markers associated with different traits using CMLM at p-value <0.001. A. DTH, B. DTM C. RS under non-stress conditions, and D. DTH, E. DTM and F. RS under drought-stress conditions. The horizontal red line represents FDR adjusted p< 0.001.

**Fig 4:**
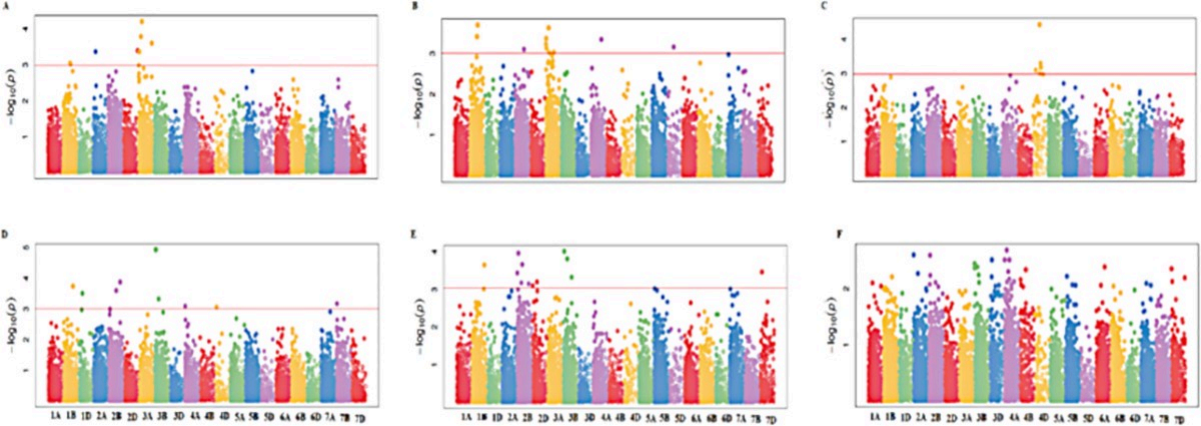
Manhattan plots showing SNP markers associated with different traits using CMLM at *p*-value <0.001. A. RB, B. SB and C. GY under non-stress conditions, and **D**. RB, **E**. SB and **F**. GY under drought-stress conditions. The horizontal red line represents FDR adjusted p< 0.001.

Phenological traits (DTH and DTM) and biomass allocation traits (RB, SB, RS and GY) were subjected to GWAS using the 15,600 SNP markers. A total of seventy-six marker traits associations (MTAs) were identified at a stringent FDR-adjusted *p* value <0.001 revealing candidate loci for each trait across different water regimes (Tables 4 and 5). There were 38 MTAs identified under each water regime (Tables 4 and 5, Fig 3). Quantile-quantile (QQ) plots (S1 Fig and S2 Fig) showed that the -log10 (p-values) for the different traits evaluated under each water regime conformed to normal distribution and were constricted enough towards expected values to account for population stratification. A total of eight, four and four significant (*P<*0.001) MTAs were detected for DTH, DTM and RS, respectively, under non-stressed condition (Table 4, Fig 3A, 3B and 3C), while there were nine and five MTAs detected for the respective traits under stressed condition (Table 5, Fig 3D and 3E). There were four markers associated with RS under drought-stress conditions (Fig 3F). A similar number of significant markers were identified for RS under non-stress and drought-stressed conditions. There were two pleiotropic markers for RB and SB detected on chromosome 1B under non-stressed conditions (Table 4, Fig 4A and 4B). A total of nine markers were observed to have significant association with grain yield, with seven occurring under non-stress conditions. Only markers on chromosome 4D were identified to have significant association with GY under non-stressed conditions (Fig 4C). Under drought-stressed conditions, three pleiotropic markers for RB and SB were identified, two were on chromosome 2B and one on chromosome 3B (Table 5, Fig 4D and 4E). There were only two markers identified for GY under drought stress (Fig 4F).

**Table 4:** SNPs significantly associated with agro-morphological traits and putative candidate genes identified in the study under non-stressed conditions.

Trait	Marker code	Chr	ChrPos	P.value	MAF	R^2^	IWGSC gene ID
DTH	M10834	5B	561671357	0,000	0,04	0,20	TRAESCS5B02G236600
	M5627	2D	568465158	0,000	0,19	0,19	TraesCS2D02G462600
	M6305	3A	56948590	0,000	0,09	0,18	TRAESCS3A02G088700
	M40	1A	2236676	0,001	0,08	0,17	TraesCS1A02G003600
	M1278	1B	379091249	0,001	0,17	0,17	
	M4469	2B	636117660	0,001	0,23	0,16	
	M9904	5A	417804694	0,001	0,21	0,16	TraesCS5A02G040700
	M576	1A	45100852	0,001	0,08	0,16	
DTM	M788	1B	81660700	0,000	0,15	0,15	TraesCS1B02G340800
	M8624	4A	114487333	0,000	0,32	0,13	TraesCS4A02G101800
	M4951	2B	753792974	0,001	0,07	0,13	TraesCS2B02G560000
	M1433	1B	554018133	0,001	0,05	0,12	
RB	M6472	3A	556260082	0,000	0,22	0,25	
	M6665	3A	702562913	0,000	0,21	0,23	
	M6529	3A	639700048	0,000	0,05	0,22	TraesCS3A02G392100
	M5573	2D	474747710	0,000	0,03	0,21	TraesCS2D02G370400
	M6500	3A	533037386	0,000	0,16	0,21	
	M3271	2A	766416023	0,000	0,38	0,21	
	M788	1B	81660700	0,001	0,15	0,19	TraesCS1B02G340800
	M1576	1B	420600926	0,001	0,19	0,19	TraesCS1B02G340800
SB	M1576	1B	420600926	0,000	0,19	0,15	TraesCS1B02G340800
	M6687	3A		0,000	0,31	0,15	
	M788	1B	81660700	0,000	0,15	0,14	TraesCS1B02G340800
	M6540	3A	662527377	0,000	0,14	0,13	TraesCS3A02G314700
	M9150	4A	22270015	0,000	0,19	0,13	
	M6660	3A	699467553	0,001	0,30	0,13	
	M12030	5D		0,001	0,03	0,12	
	M6088	3A	7019713	0,001	0,12	0,12	
	M3933	2B	154602689	0,001	0,18	0,12	
	M6638	3A	695160174	0,001	0,47	0,12	
	M6697	3A	711289967	0,001	0,21	0,12	
RS	M1566	1B	592833254	0,001	0,31	0,17	
	M7197	3B		0,001	0,08	0,16	
	M7187	3B	825539318	0,001	0,24	0,16	TraesCS3B02G606400
	M5066	2B	595286130	0,001	0,28	0,15	
GY	M9776	4D	442739513	0,000	0,35	0,19	TraesCS4D02G272500
	M9769	4D	401082716	0,001	0,07	0,13	TraesCS4D02G238900
	M9806	4D	250840711	0,001	0,20	0,12	
	M9759	4D	336833285	0,001	0,38	0,12	TraesCS4D02G193400
	M9756	4D	220375714	0,001	0,05	0,11	
	M9813	4D	36740586	0,001	0,37	0,11	

Chr = chromosome, ChrPos = chromosome position, MAF = minor allele frequency

**Table 5:** SNPs significantly associated with agro-morphological traits and putative candidate genes identified in the study under drought stress conditions.

	Marker code	Chr	ChrPos	P.value	MAF	R2	IWGSC gene ID
DTH	M5902	2D	606129207	0,000	0,27	0,36	TraesCS2D02G514100
	M1626	1B	400908048	0,000	0,07	0,36	
	M892	1B	12505139	0,000	0,16	0,36	TraesCS1B02G032700
	M7199	3B	157603272	0,001	0,06	0,35	TraesCS3B02G061700
	M8019	3B	765843802	0,001	0,06	0,35	TraesCS3B02G523300
	M8011	3B	533130365	0,001	0,43	0,35	TraesCS3B02G337500
	M7428	4A	733792535	0,001	0,16	0,35	TraesCS4A02G161100
	M7089	3B	22917259	0,001	0,45	0,34	TraesCS3B02G045600
	M7970	3B	95564668	0,001	0,23	0,34	TraesCS3B02G154000
DTM	M3664	2B	59764104	0,000	0,03	0,34	
	M13563	6B	124069487	0,000	0,02	0,33	
	M3989	2B	126899677	0,000	0,18	0,31	TraesCS2B02G201000
	M3717	2B	77176639	0,000	0,20	0,30	TraesCS2B02G114000
	M9936	5A	37492810	0,000	0,20	0,30	
RB	M7970	3B	95564668	0,000	0,23	0,32	TraesCS3B02G154000
	M4551	2B	565077064	0,000	0,37	0,26	TraesCS2B02G398200
	M1378	1B	471174490	0,000	0,32	0,26	TraesCS1B02G268300
	M4676	2B	595508003	0,000	0,24	0,25	TraesCS2B02G528800
	M2116	1D	472550886	0,000	0,33	0,25	TraesCS1D02G276600
	M8061	3B	785499330	0,000	0,43	0,24	TraesCS3B02G550700
	M15552	7B	99953334	0,001	0,22	0,23	TraesCS7B02G377800
	M9282	4A	688382396	0,001	0,19	0,23	
	M9734	4D	79280510	0,001	0,12	0,22	
	M7199	3B	157603272	0,001	0,06	0,35	TraesCS3B02G061700
SB	M7970	3B	95564668	0,000	0,23	0,32	TraesCS3B02G154000
	M7199	3B	157603272	0,001	0,06	0,35	TraesCS3B02G061700
	M7089	3B	22917259	0,000	0,45	0,31	TraesCS3B02G045600
	M3717	2B	77176639	0,000	0,20	0,30	TraesCS2B02G114000
	M1785	1B	646178482	0,000	0,13	0,30	
	M1511	7D	68	0,000	0,37	0,29	TraesCS7D02G150700
	M4368	2B	459249657	0,000	0,05	0,29	TraesCS2B02G321800
	M7428	3B	303590858	0,000	0,42	0,28	
	M5706	2D	591603469	0,001	0,19	0,28	TraesCS2D02G494600
	M4110	2B	384320443	0,001	0,04	0,28	
	M4551	2B	565077064	0,001	0,37	0,28	TraesCS2B02G398200
	M5909	2D	615707186	0,001	0,20	0,27	
	M11177	5B	2141663	0,001	0,47	0,27	
	M1798	1B	646777010	0,001	0,01	0,27	
	M14627	7A	670757369	0,001	0,24	0,27	TraesCS7A02G167900
RS	M12045	6A	663760681	0,000	0,30	0,15	TraesCS6A02G005500
	M3562	2B	419250206	0,001	0,34	0,13	
	M9433	4A	743768873	0,001	0,37	0,13	
	M9434	4D	16502547	0,001	0,21	0,13	
	M3559	2B	1204170	0,001	0,32	0,13	
GY	M8680	4A	157557592	0,001	0,12	0,16	TraesCS4A02G124400
	M2966	2A	705340913	0,001	0,04	0,16	TraesCS2A02G456400

Chr = chromosome, ChrPos = chromosome position, MAF = minor allele frequency

### Putative candidate gene analysis and expression data

**Fig 5:**
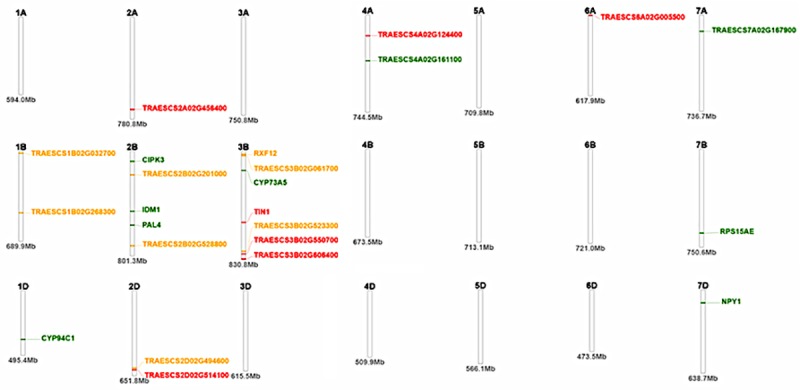
Physical map of the wheat genome showing the positions of the identified genes localized with the some of the SNP markers. TRAESCS2B02G321800 = IDM1, TRAESCS2B02G114000 = CIPK3, TRAESCS2B02G398200 = PAL4, TRAESCS3B02G154000 = CYP73A5, TRAESCS2D02G370400 = ABCG11, TRAESCS4D02G238900 = WAKL21, TRAESCS5B02G236600 = AMY1, TRAESCS7B02G377800 = RPS15AE, TRAESCS1D02G276600 = CYP94C1, TRAESCS7D02G150700 = NPY1, TRAESCS4D02G272500 = PSAT2, TRAESCS1B02G340800 = FH6, TRAESCS3B02G045600 = RXF12, TRAESCS3B02G337500 = TIN1.

The physical genetic map shows that a number of the markers were co-localized, especially on chromosomes 2B, 2D, 3B, 4A and 4D (Fig 5). Co-localization of genes was observed on chromosome 2D and 3B at positions 591.6 and 785Mb, respectively. DTH had two genes TraesCS2D02G462600 and TraesCS2D02G514100 in close proximity on the 2D chromosome and TraesCS2D02G370400 overlapping the significant MTAs for RB. The two pleiotropic markers for RB and SB detected on chromosome 1B flanked a region overlapping the TraesCS1B02G340800 (Table 5). One of the identified marker on chromosome 2B covered a region overlapping the gene TraesCS2B02G398200 while the markers on chromosome 3B overlapped gene TraesCS3B02G154000 and TraesCS3B02G061700. On chromosome 3B, there was co-localization of genes overlapping significant MTAs for DTH, RB and RS. Interestingly, RB and SB shared common loci and pleiotropic markers showing that they are genetically highly correlated. The common loci for RB and SB on chromosome 1B was associated with gene FH6 (position 568.75Mb). The other pleiotropic loci on chromosomes 2B overlapping genes PAL4 at positions 565.06Mb, and on chromosome 3B in the region coding for TraesCS3B02G061700 (position 33.95Mb) and CYP73A5 (at 146.55Mb).

### Linkage disequilibrium among the markers

**Fig 6:**
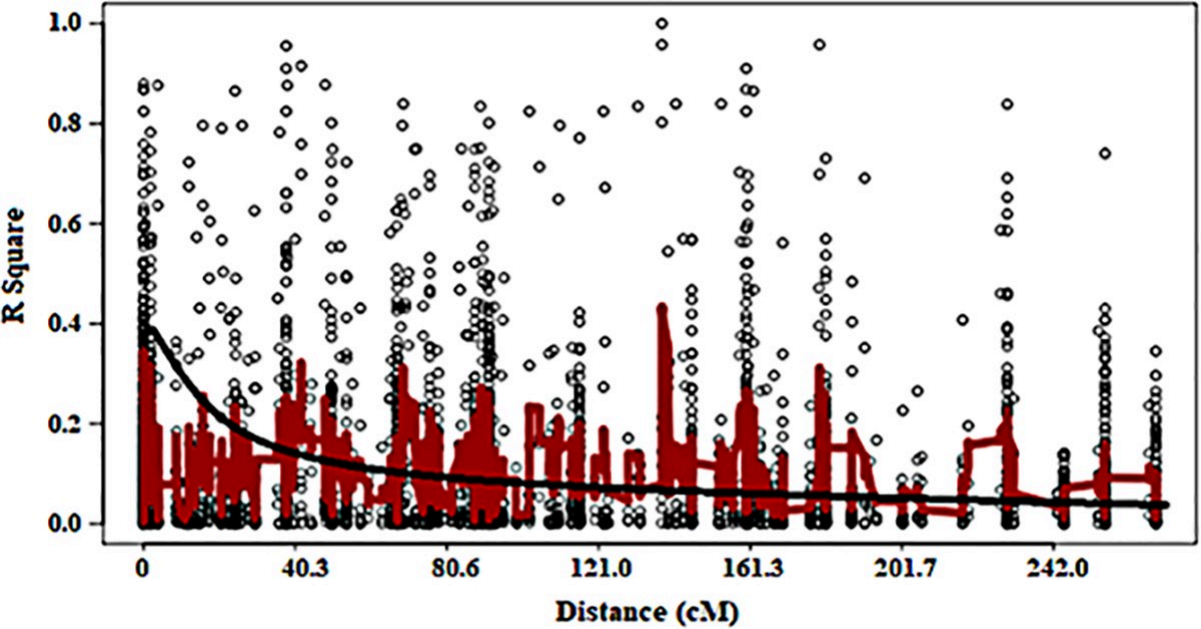
Linkage disequilibrium (R^2^) plot of all the 15,600 SNP markers across genomes in 97 wheat genotypes used in the mapping study.

**Fig 7:**
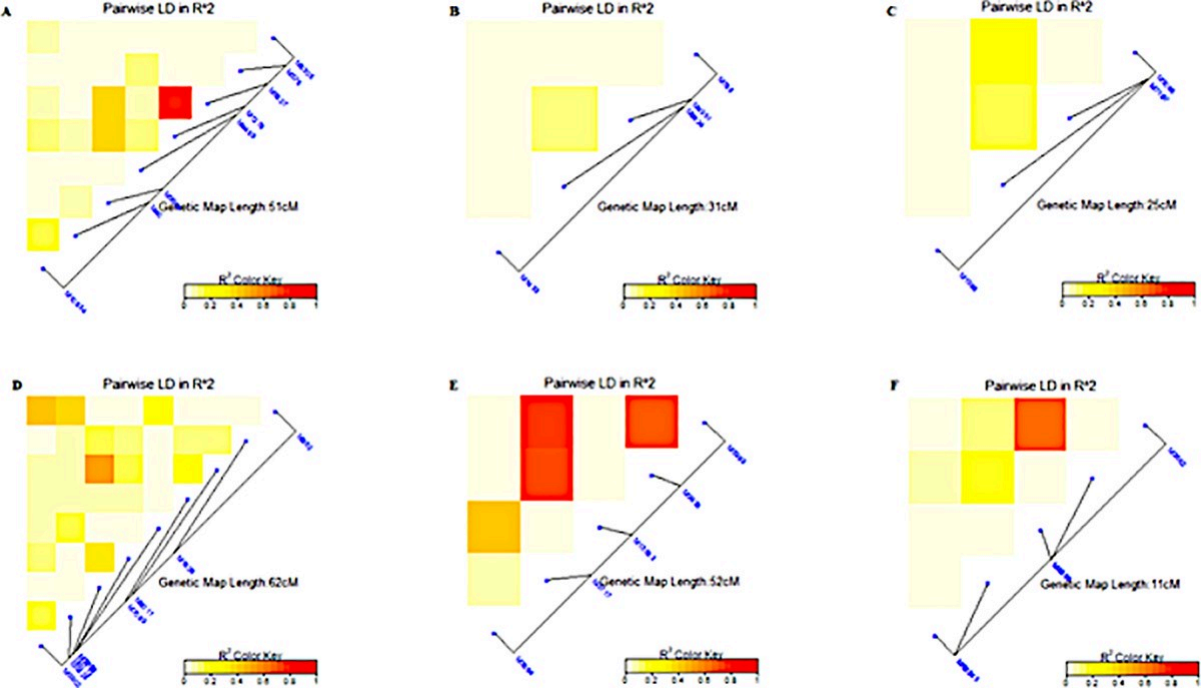
Summary of the local LD among markers with significant MTAs for different traits. A. DTH, B. DTM and C. RS under non-stress conditions and D. DTH, E. DTM and F. RS under drought-stress conditions. The R^2^ color key indicates the degree of significant association.

**Fig 8:**
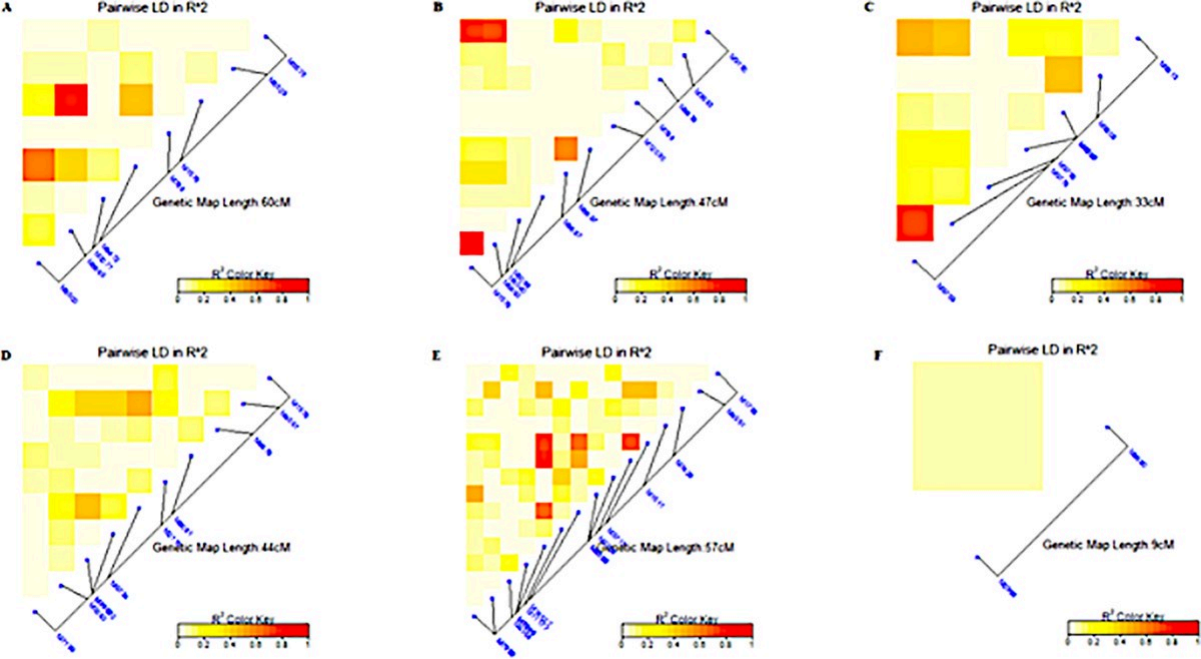
Summary of the local LD among markers with significant MTAs for different traits. A. RB, B. SB and C. GY under non-stress conditions and D. RB, E. SB and F. GY under drought-stress conditions. The R^2^ color key indicates the degree of significant association.

The markers exhibited a linkage disequilibrium decay across the whole genome with an estimated threshold value of R^2^ = 0.38 at about 50Mbp based on the 95^th^ percentile of the distribution (Fig 6). The linkage disequilibrium (LD) analysis was conducted on SNP markers with significant association with a particular trait under each water regime. The LD ranged from very weak correlation (r<0.20, p<0.001) for SNP markers associated with DTM under non-stress conditions (Fig 7A–7F) to strong correlations (r>0.80, p<0.001) for SB under well-watered conditions (Fig 8A–8F). For RB, there were two markers M1576 and M6665, which exhibited linkage disequilibrium (r>0.080, p<0.001) under non-stress conditions (Fig 8A). Three markers M1576, M6660 and M9150 were in disequilibria for SB under non-stress and they were within 47cM (Fig 8B). For GY, there were two markers in linkage disequilibria (r>0.80, p<0.001) identified under non-stress conditions spanning a 33cM length (Fig 8C). None of the significant MTAs showed high level of LD for RB under drought stress conditions (Fig 8D). There were suggestions of distinct haplotypes for SB under drought stress (Fig 8E). For SB under drought stress, the markers exhibited low to high LD over 57cM (Fig 8E) while the significant markers for GY had low LD (Fig 8F).

## Discussion

### Phenotypic variability of germplasm and environmental response

Understanding biomass allocation in wheat could provide an opportunity and an alternative approach to developing drought tolerant cultivars that can also sequester relatively more C for soil remediation. The wheat genotypes evaluated in this study exhibited wide genetic variation for agronomic and biomass accumulation traits (roots, shoot and grain) (Tables 1 and 2). The wide genetic variation was expected since the population included genotypes from CIMMYT, local accessions and temperate adapted cultivars. Biomass accumulation in roots, shoot and grains was significantly reduced by 32, 30 and 48%, respectively, under drought stress confirming that biomass accumulation has phenotypic plasticity. This plasticity could be exploited in drought tolerance breeding of wheat to mitigate water scarcity [49]. The variance components, heritability and genetic correlations of the traits were reported in [50]. The traits exhibited different levels of heritability, with RB (H = 78%) and SB (H = 64%) having higher heritability estimates than RS (H = 28%) and GY (H = 17%). However, the lower heritability estimates observed under stressed condition could reduce selection efficiency [49] and may impact negatively on QTL detection [51].

### Population structure and linkage disequilibrium

The population structure and principal component analyses revealed that the genotypes could be divided into two distinct major clusters (Figs 1 and 2). Following the method of [52], the K value with the highest ΔK-value confirms the number of appropriate clusters for that population [16; 35; 53]. The two clusters identified separated the genotypes into one cluster composed mainly of the genotypes from the CIMMYT heat tolerant nursery while the other clusters consisted of drought tolerant and local checks. However, the structure analysis also identified the presence of admixtures and the two clusters could be further delineated into six sub-clusters. The genotypes form part of a training population that can be used to develop and optimize a model for predicting genomic estimated breeding value (GEBV) since it has now been phenotyped and genotyped [54]. The mean fixation indices (F_st_) associated with the six clusters ranged between 0.45 and 0.85, indicating a potentially high level of differentiation among the clusters although within cluster variation was low as shown by the heterozygosity values ranging between 0.09 and 0.24. Based on the mean fixation indices and the genetic distances, the genotypes form part of a training population that can be used to develop and optimize a model for predicting genomic estimated breeding value since it has now been phenotyped and genotyped [54]. However, these F_st_ values should be used cautiously in analyzing diversity or differentiation as these statistics are often misconstrued [55]. The resultant population structure and genetic distances between pairs of clusters observed in this study also confirmed the existence of admixtures and kinship. The admixtures and kinship patterns observed were attributed to sharing of common parentage among some of the genotypes. For instance, 17 out of the 32 genotypes including genotypes BW124, BW147, BW151 and BW159 in sub-cluster 1 shared a common parent CGSS05B00258T-099TOPY. Parent CROC_1/AE.SQUARROSA was common for 4 genotypes LM79, LM81, LM81 and LM90 in sub-cluster 2 while WBLL1 and PASTOR were common parents a considerable number of genotypes. The use of a small number of elite varieties exhibiting desirable traits and routinely crossed to fix the desirable alleles is a standard practice in developing modern wheat cultivars [56], which contributes to narrowing of genetic diversity.

### Marker-trait associations and putative genes

The population structure of the panel, the variance components, heritability and genetic correlations for the phenotypic traits confirmed that the panel of genotypes was suitable for use in a genome wide association study involving yield and biomass traits. The use of a diverse panel of genotypes can provide more valuable inference compared to bi-parental populations [57] by taking advantage of maximum allelic diversity [58; 59].

The identified 77 significant markers associated with the phenological and biomass allocation traits included 36 that were detected on the B genome similar to other studies that previously detected significant markers for root and shoot biomass on this genome [57; 60]. [57] found extremely rare haplotype variants that increased root growth on chromosome 5B, while [60] reported significant SNPs on 1A, 2A, 3B, 5B, 6A, and 7B for root dry weight with the major two QTLs being on 1A and 5B. The remaining significant SNPs detected in this study, which have not been reported previously could be novel alleles important for influencing biomass allocation patterns in wheat. The major QTLs reported by [60] were found using seedling data unlike in this study, which used phenotypic data collected on mature plants. This is useful since selection at early stages may not reflect trait performance at later growth stages, particularly, for traits that are relevant for drought tolerance and C sequestration.

Significant pleiotropic loci were detected on the B genome for root and shoot biomass, showing that root and shoot biomass have common and distinct genomic loci. Root and shoot biomass shared an association region on chromosomes 1B, 2B and 3B which suggest that this could be the basis for their high genetic correlation as reported by [50]. The common loci for RB and SB on chromosome 1B was associated with gene FH6 (TraesCS1B02G340800 gene), which is known for signaling pathways in root lateral meristem and shoot apex development [61]. The identified marker on chromosome 2B covered a region overlapping the gene PAL4, known for upregulating protein for stress response and stem elongation [62]. The identification of a putative gene TraesCS3B02G061700 on chromosome 3B for RB and SB and its co-localization with gene RXF12 for DTH is an indication of a strong physical linkage among these traits. The putative gene TraesCS3B02G061700 is known to be actively involved in the photosystem I [63], which could explain its influence on biomass accumulation. The RXF12 gene has been implicated in the defense mechanism against drought and heat stress in *Arabidopis* [64] and their suggested strong linkage could assist in simultaneous selection for high root and shoot biomass and drought tolerance in wheat. The detection of common SNPs for root and shoot biomass on the B genome under drought stress suggests that it carries the critical loci controlling for root biomass and possibly drought tolerance mechanisms [58] and provides an opportunity for effective simultaneous improvement using the overlapping markers. In reality, many complex traits exhibit linkage and selection of pleiotropic genes has potential to cause major simultaneous changes in the traits [65; 66]. However, there is a concern that increasing below ground biomass might negatively affect other economic traits due to undesirable linkage drag associated with unfavorable pleiotropy. [67] asserted that simultaneous improvement of root and above ground traits will only be possible if they have common and distinct genomic loci that can be manipulated independently or simultaneously. The marker was found to be in LD with other markers associated with DTH, SB and RB indicating tight linkage, which could provide opportunities for biomass allocation improvement in wheat. [57] suggested that unfavorable linkage drag between negatively correlated traits can be overcome by identifying rare recombinant genes.

The current association study identified two markers significant for GY under drought stressed conditions, of which TRAESCS4A02G124400 was reported to prolonged seed dormancy, caused male sterility, and dwarfism in rice [68]. Under non-stress conditions, two putative genes were identified. The gene TRAESCS4D02G238900 possibly affected grain yield accumulation through indirect effects on 100-seed weight, seed length and the regulation of cytokins [69] while TRAESCS4D02G238900 regulated leaf senescence [70]. The indirect effects on grain yield via up- or down-regulation of cytokins or control of leaf senescence has been established in wheat [71]. The low number of observed MTAs for GY under drought-stressed condition was consistent with [16] and [72] who also found reduced number of MTAs for GY under drought stress in wheat. Grain yield is highly influenced by genotype by environment interaction, which could have negatively impacted the ability to detect the associated markers under stressed conditions. The identified MTAs under non-stressed conditions are useful for future marker-assisted selection. The genomic information obtained here would be useful to improve accuracy in estimating the breeding value of related genotypes.

Overall, across all genomes and markers, an LD of 0.38 occurring at 5cM indicated that the LD decay occurred at relatively shorter distances, which can be attributed to narrow genetic variation due to repeated backcrossing to a limited number of elite breeding lines. A substantial number of SNPs that were significantly associated with RB, SB and GY occurred on genetic regions spanning between 9 and 60cM at an average LD of 0.40, showing the possibility of tight to moderate linkage. Similarly, [73] found an average LD of 0.2 extending over 2-3cM while there were some loci extending between 25 and 41cM with LD >0.7. [74] found moderate (<20cM) and loose (>50cM) inter-chromosomal linkage in closely related durum (*Triticum durum* Desf.) using microsatellite markers. The observed markers with non-significant LD is not unique given that other studies reported them as a result of possible admixtures of the genotypes [75; 76].

## Conclusions

The use of a diverse population of wheat genotypes with different pedigrees allowed for detection of 77 MTAs for days to heading and maturity and, biomass allocation to roots, shoots and grain yield. The identified markers such as M788, M1576 and M7199 for root biomass can be used in marker-assisted selection to improve the root system of wheat. These markers are useful in breeding for drought tolerance and C sequestration. The seven pleiotropic markers for root and shoot biomass will enable simultaneous selection for above and below ground biomass suggesting that drought tolerance and C sequestration are tightly linked. The identified MTAs on chromosomes 1B, 2B, 3A, 4D and 7A that have not been previously reported could provide novel genes for wheat breeding. This study provides a foundation for marker-assisted breeding for biomass allocation drought tolerance and C sequestration in wheat. Validation of the identified markers using a diverse and large size or bi-parental population, and using tissue and stage specific gene expression data from RNASeq would be required before embarking on a large scale breeding program.

## Supporting information

S1 Table List of genotypes and their pedigree used in the study. (DOCX)

S2 Table Genetic distances between different clusters obtained from structure analysis of 99 wheat genotypes and a triticale accession. (DOCX)

S3 Table GAPIT Results for DTH under non stress. (CSV)

S4 Table GAPIT Results for DTH under drought. (CSV)

S5 Table GAPIT Results for DTM under non stress. (CSV)

S6 Table GAPIT Results for DTM under drought. (CSV)

S7 Table GAPIT Results for RS under non stress. (CSV)

S8 Table GAPIT Results for RS under drought. (CSV)

S9 Table GAPIT Results for RB under non stress. (CSV)

S10 Table GAPIT Results for RB under drought. (CSV)

S11 Table GAPIT Results for SB under non stress. (CSV)

S12 Table GAPIT Results for SB under drought. (CSV)

S13 Table GAPIT Results for GY under non stress. (CSV)

S14 Table GAPIT Results for GY under drought. (CSV)

S1 Fig Quantile-Quantile plots indicating the normality of data for different traits (A) DTH, (B) DTM and (C) RS under non-stress conditions and (D) DTH, (E) DTM and (F) RS under drought-stress conditions.
(TIF)

S2 Fig Quantile-Quantile plots indicating the normality of data for different traits (A) RB, (B) SB and (C) GY under non-stress conditions and (D) RB, (E) SB and (F) GY under drought-stress conditions.
(TIF)
