# Supplementary material for: Genome-wide association study of drought tolerance and biomass allocation in wheat
Source: PLoS One. 2019 Dec 4;14(12):e0225383. doi: 10.1371/journal.pone.0225383 (PMC6892492; doi:10.1371/journal.pone.0225383)
Supplement: S1 Table — (DOCX) [file pone.0225383.s001.docx]

Supplementary Table 1 List of genotypes and their pedigree used in the study

| **ENTRY CODE** | **PEDIGREE** |
| --- | --- |
|  | **Genotypes from CIMMYT Heat Stress Tolerance Nursery** |
| LM01 | ACHTAR*3//KANZ/KS85-8-5/4/MILAN/KAUZ//PRINIA/3/BAV92/5/MILAN/KAUZ//PRINIA/3/BAV92 |
| LM12 | SOKOLL/ROLF07 |
| LM14 | MILAN/KAUZ//PRINIA/3/BAV92/4/WBLL1*2/KUKUNA |
| LM15 | RL6043/4*NAC//PASTOR/3/BAV92/4/ATTILA/BAV92//PASTOR |
| LM16 | PASTOR*2/BAV92/3/FRET2/KUKUNA//FRET2 |
| LM17 | ESDA/KKTS |
| LM18 | GOUBARA-1/2*SOKOLL |
| LM19 | SOKOLL*2/4/CHEN/AEGILOPS SQUARROSA (TAUS)//FCT/3/STAR |
| LM20 | PBW343 |
| LM21 | PRL/2*PASTOR |
| LM22 | MUNAL #1 |
| LM23 | QUAIU |
| LM24 | WBLL1*2/BRAMBLING |
| LM25 | WHEAR//2*PRL/2*PASTOR |
| LM26 | ATTILA*2/PBW65//TAM200/TUI |
| LM27 | YUNMAI 48//2*WBLL1*2/KURUKU |
| LM28 | ATTILA/3*BCN//BAV92/3/TILHI/4/SHA7/VEE#5//ARIV92 |
| LM29 | PRL/2*PASTOR*2//SKAUZ/BAV92 |
| LM30 | C80.1/3*BATAVIA//2*WBLL1/3/ATTILA/3*BCN*2//BAV92/4/WBLL1*2/KURUKU |
| LM31 | ATTILA*2/HUITES//FINSI/3/ATTILA*2/PBW65 |
| LM32 | ATTILA*2//CHIL/BUC*2/3/KUKUNA |
| LM33 | ATTILA*2/PBW65//KACHU |
| LM35 | WBLL1//UP2338*2/VIVITSI |
| LM36 | WBLL1*2/4/SNI/TRAP#1/3/KAUZ*2/TRAP//KAUZ/5/KACHU |
| LM37 | KACHU/SAUAL |
| LM38 | SAUAL/3/MILAN/S87230//BAV92 |
| LM39 | ATTILA/3*BCN//BAV92/3/TILHI/5/BAV92/3/PRL/SARA//TSI/VEE#5/4/CROC_1/AE.SQUARROSA (224)//2*OPATA |
| LM40 | WBLL1*2/VIVITSI/6/CNDO/R143//ENTE/MEXI_2/3/AEGILOPS SQUARROSA (TAUS)/4/WEAVER/5/2*JANZ |
| LM41 | C80.1/3*BATAVIA//2*WBLL1/5/REH/HARE//2*BCN/3/CROC_1/AE.SQUARROSA (213)//PGO/4/HUITES |
| LM42 | TRCH/5/REH/HARE//2*BCN/3/CROC_1/AE.SQUARROSA (213)//PGO/4/HUITES |
| LM43 | ROLF07*2/6/PVN//CAR422/ANA/5/BOW/CROW//BUC/PVN/3/YR/4/TRAP#1 |
| LM44 | ROLF07/TUKURU/5/WBLL1*2/4/YACO/PBW65/3/KAUZ*2/TRAP//KAUZ |
| LM46 | FRET2/KUKUNA//FRET2/3/PARUS/5/FRET2*2/4/SNI/TRAP#1/3/KAUZ*2/TRAP//KAUZ |
| LM47 | FRET2/KUKUNA//FRET2/3/YANAC/4/FRET2/KIRITATI |
| LM48 | FRET2/KUKUNA//FRET2/3/PASTOR//HXL7573/2*BAU/5/FRET2*2/4/SNI/TRAP#1/3/KAUZ*2/TRAP//KAUZ |
| LM49 | TRCH/SRTU//KACHU |
| LM50 | HUW234+LR34/PRINIA*2//SNLG |
| LM51 | HUW234+LR34/PRINIA*2//YANAC |
| LM52 | HUW234+LR34/PRINIA*2//WHEAR |
| LM54 | PBW343*2/KUKUNA*2//KITE |
| LM55 | PBW343*2/KUKUNA//PARUS/3/PBW343*2/KUKUNA |
| LM56 | PBW343*2/KUKUNA*2//YANAC |
| LM57 | PBW343*2/KUKUNA//SRTU/3/PBW343*2/KHVAKI |
| LM58 | ATTILA*2/PBW65/6/PVN//CAR422/ANA/5/BOW/CROW//BUC/PVN/3/YR/4/TRAP#1/7/ATTILA/2*PASTOR |
| LM59 | FRET2/KUKUNA//FRET2/3/WHEAR/4/FRET2/TUKURU//FRET2 |
| **Table S1 Continued** | |
| **ENTRY CODE** | **PEDIGREE** |
| LM60 | ALD/CEP75630//CEP75234/PT7219/3/BUC/BJY/4/CBRD/5/TNMU/PF85487/6/PBW343*2/KUKUNA/7/CNO79//PF70354/MUS/3/PASTOR/4/BAV92 |
|  | **Genotypes from CIMMYT Drought Stress Tolerance Nursery** |
| LM71 | BABAX/3/PRL/SARA//TSI/VEE#5/4/CROC_1/AE.SQUARROSA (224)//2*OPATA |
| LM72 | BABAX/3/PRL/SARA//TSI/VEE#5/4/WBLL1 |
| LM75 | BUC/MN72253//PASTOR |
| LM76 | MILAN/KAUZ//PRINIA/3/BABAX |
| LM77 | CNDO/R143//ENTE/MEXI_2/3/AEGILOPS SQUARROSA (TAUS)/4/WEAVER/5/2*FRAME |
| LM79 | CROC_1/AE.SQUARROSA (205)//BORL95/3/KENNEDY |
| LM80 | CROC_1/AE.SQUARROSA (205)//KAUZ/3/SLVS |
| LM81 | CROC_1/AE.SQUARROSA (224)//2*OPATA/3/2*RAC655 |
| LM82 | HD30/5/CNDO/R143//ENTE/MEXI75/3/AE.SQ/4/2*OCI |
| LM83 | PASTOR/3/VEE#5//DOVE/BUC |
| LM84 | SRN/AE.SQUARROSA (358)//MILAN/SHA7 |
| LM85 | SW94.60002/4/KAUZ*2//DOVE/BUC/3/KAUZ/5/SW91-12331 |
| LM86 | CHAM 6 |
| LM90 | CROC_1/AE.SQUARROSA (205)//BORL95/3/KENNEDY-2 |
| LM91 | FRTL/CMH83.2517 |
| LM93 | PASTOR/FLORKWA.1//PASTOR |
| LM96 | ALTAR 84/AE.SQ//2*OPATA/3/PIFED |
| LM97 | KRICHAUFF/2*PASTOR |
| LM98 | KABY//2*ALUBUC/BAYA |
| LM99 | ALTAR 84/AEGILOPS SQUARROSA (TAUS)//OCI/3/VEE/MJI//2*TUI |
| LM100 | SW89.5277/BORL95//SKAUZ |
|  | **Genotypes from CIMMYT Heat Stress Tolerance Nursery** |
| BW28 | CMSA05Y01011T-040M-040ZTP0Y-040ZTM-040SY-14ZTM-03Y-0B |
| BW48 | CMSA04M00346S-040ZTP0Y-040ZTM-040SY-27ZTM-04Y-0B |
| BW49 | CMSA04M00346S-040ZTP0Y-040ZTM-040SY-28ZTM-01Y-0B |
| BW58 | CMSA04M00067S-040ZTB-040ZTY-040ZTM-040SY-2ZTM-02Y-0B |
| BW63 | CMSA04M01020T-050Y-040ZTP0M-040ZTY-040ZTM-040SY-5ZTM-03Y-0B |
| BW71 | CMSA05Y00325S-040ZTP0Y-040ZTM-040SY-7ZTM-01Y-0B |
| BW103 | CMSS05B00581S-099Y-099M-099Y-099ZTM-2WGY-0B |
| BW111 | CMSS05B00663S-099Y-099M-099Y-099ZTM-13WGY-0B |
| BW116 | CMSS05B00742S-099Y-099M-099Y-099ZTM-5WGY-0B |
| BW124 | CGSS05B00153T-099TOPY-099M-099NJ-22WGY-0B |
| BW127 | CGSS05B00162T-099TOPY-099M-099Y-099ZTM-15WGY-0B |
| BW128 | CGSS05B00162T-099TOPY-099M-099NJ-13WGY-0B |
| BW129 | CGSS05B00162T-099TOPY-099M-099NJ-099NJ-7WGY-0B |
| BW141 | CGSS05B00243T-099TOPY-099M-099NJ-099NJ-1WGY-0B |
| BW142 | CGSS05B00243T-099TOPY-099M-099NJ-099NJ-2WGY-0B |
| BW145 | CGSS05B00253T-099TOPY-099M-099Y-099ZTM-8WGY-0B |
| BW147 | CGSS05B00256T-099TOPY-099M-099NJ-099NJ-5WGY-0B |
| BW148 | CGSS05B00258T-099TOPY-099M-099Y-099ZTM-3WGY-0B |
| BW149 | CGSS05B00258T-099TOPY-099M-099Y-099ZTM-11WGY-0B |
| BW150 | CGSS05B00258T-099TOPY-099M-099Y-099ZTM-12WGY-0B |
| BW151 | CGSS05B00258T-099TOPY-099M-099Y-099ZTM-13WGY-0B |
| BW152 | CGSS05B00258T-099TOPY-099M-099NJ-1WGY-0B |
| BW157 | CGSS05B00261T-099TOPY-099M-099NJ-099NJ-8WGY-0B |
| **Table S1 Continued** | |
| **ENTRY CODE** | **PEDIGREE** |
| BW159 | CGSS05B00290T-099TOPY-099M-099NJ-099NJ-7WGY-0B |
| BW162 | CGSS05B00304T-099TOPY-099M-099NJ-099NJ-3WGY-0B |
|  | **Local Checks** |
| LM70 | Check |
| BW80 | Check |
| BW100 | Check |
| BW120 | Check |
| BW140 | Check |
|  | **Temperate checks** |
| Arenza | Check |
| Sossognon | Check |
| Triticale | Check |
