# Supplementary material for: Genome-wide association study of drought tolerance and biomass allocation in wheat
Source: PLoS One. 2019 Dec 4;14(12):e0225383. doi: 10.1371/journal.pone.0225383 (PMC6892492; doi:10.1371/journal.pone.0225383)
Supplement: S2 Table — (DOCX) [file pone.0225383.s002.docx]

**Supplementary Table 2** Genetic distances between different clusters obtained from structure analysis of 99 wheat genotypes and a triticale accession

| Clusters | 1 | 2 | 3 | 4 | 5 | 6 |
| --- | --- | --- | --- | --- | --- | --- |
| 1 | - |  |  |  |  |  |
| 2 | 0.3835 | - |  |  |  |  |
| 3 | 0.3272 | 0.1772 | - |  |  |  |
| 4 | 0.3827 | 0.2236 | 0.2102 | - |  |  |
| 5 | 0.4009 | 0.2211 | 0.1591 | 0.2346 | - |  |
| 6 | 0.3327 | 0.1771 | 0.1539 | 0.1794 | 0.1936 | - |
