# Supplementary figures and images for: Genome-wide association study of drought tolerance and biomass allocation in wheat
Source: PLoS One. 2019 Dec 4;14(12):e0225383. doi: 10.1371/journal.pone.0225383 (PMC6892492; doi:10.1371/journal.pone.0225383)

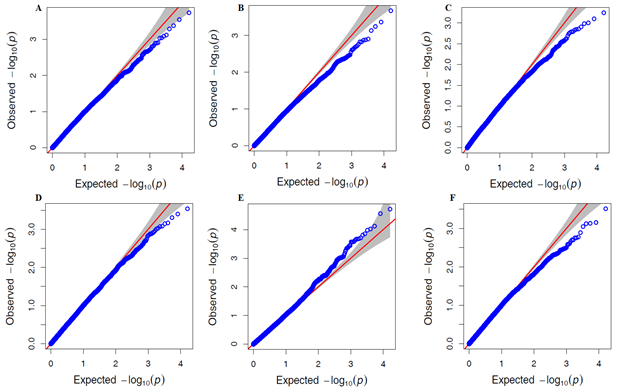

Supplement: S1 Fig — Quantile-Quantile plots indicating the normality of data for different traits (A) DTH, (B) DTM and (C) RS under non-stress conditions and (D) DTH, (E) DTM and (F) RS under drought-stress conditions. (TIF) [file pone.0225383.s015.tif]

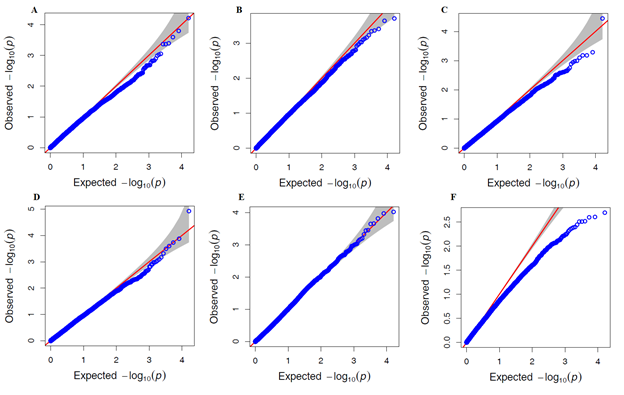

Supplement: S2 Fig — Quantile-Quantile plots indicating the normality of data for different traits (A) RB, (B) SB and (C) GY under non-stress conditions and (D) RB, (E) SB and (F) GY under drought-stress conditions. (TIF) [file pone.0225383.s016.tif]
